# Imaging of Classic Dalén Fuchs Nodules in Sympathetic Ophthalmia With Spectral Domain OCT

**DOI:** 10.7759/cureus.20876

**Published:** 2022-01-02

**Authors:** Kaleem Ahmed, M. A. Rehman Siddiqui, Humera Sarwar

**Affiliations:** 1 Medicine, Aga Khan Univeristy, Karachi, PAK; 2 Ophthalmology, Aga Khan University, Karachi, PAK; 3 Medicine, Peninsula College of Medicine and Dentistry, Exeter, GBR

**Keywords:** sympathetic ophthalmia, spectral domain oct, dalen fuchs

## Abstract

Sympathetic ophthalmia (SO) is a rare, severe condition that typically presents as bilateral diffuse granulomatous uveitis secondary to trauma in one eye. The variability of symptoms requires that diagnosis depends heavily on the correlation of these symptoms with relevant imaging. Visualisation of characteristic nodules seen between the Bruchs membrane and the retinal pigment epithelium, and the presence of Dalén Fuchs nodules, can be diagnostic when coupled with the clinical findings. This report discusses the use of spectral domain optical coherence tomography (OCT) to indicate the presence of Dalén Fuchs nodules, which have previously not been identified on OCT in a confirmed case of SO.

## Introduction

Sympathetic ophthalmia (SO) is described by Castiblanco et al. [1] as an inflammatory disorder of the eye often following penetrating ocular trauma or surgery. The etiology is not clear but SO is thought to arise from an autoimmune inflammatory process resulting in diffuse bilateral uveitis which begins in the previously traumatized, inciting eye, progressing to the remaining, sympathizing eye. Symptoms can range from decreased visual acuity, epiphora, photophobia, floaters, pain, and photopsia to significant visual loss [1]. Anteriorly, mutton-fat keratic precipitates and synechiae may be seen coupled with increased intraocular pressure. Reynard et al. [2] explained that posteriorly, Dalén Fuchs nodules form between Bruch's membrane and the retinal pigment epithelium. The nodules are indicated by the presence of yellow-white subretinal pigment epithelium lesions. Their presence is a characteristic pathological finding in SO. However, they are also seen in other conditions such as Vogt-Koyanagi-Harada syndrome (VKH) [2]. Histology reveals a nodular mass on Bruch's membrane consisting of depigmented epithelial cells and lymphocytes capped by retinal pigment epithelium [3] as found by Arevalo et al.

## Case presentation

A 60-year-old male patient who had undergone past surgery for retinal detachment repair and cataract removal in his right eye, presented with recent onset photopsia in his left eye. 

**Figure 1:**
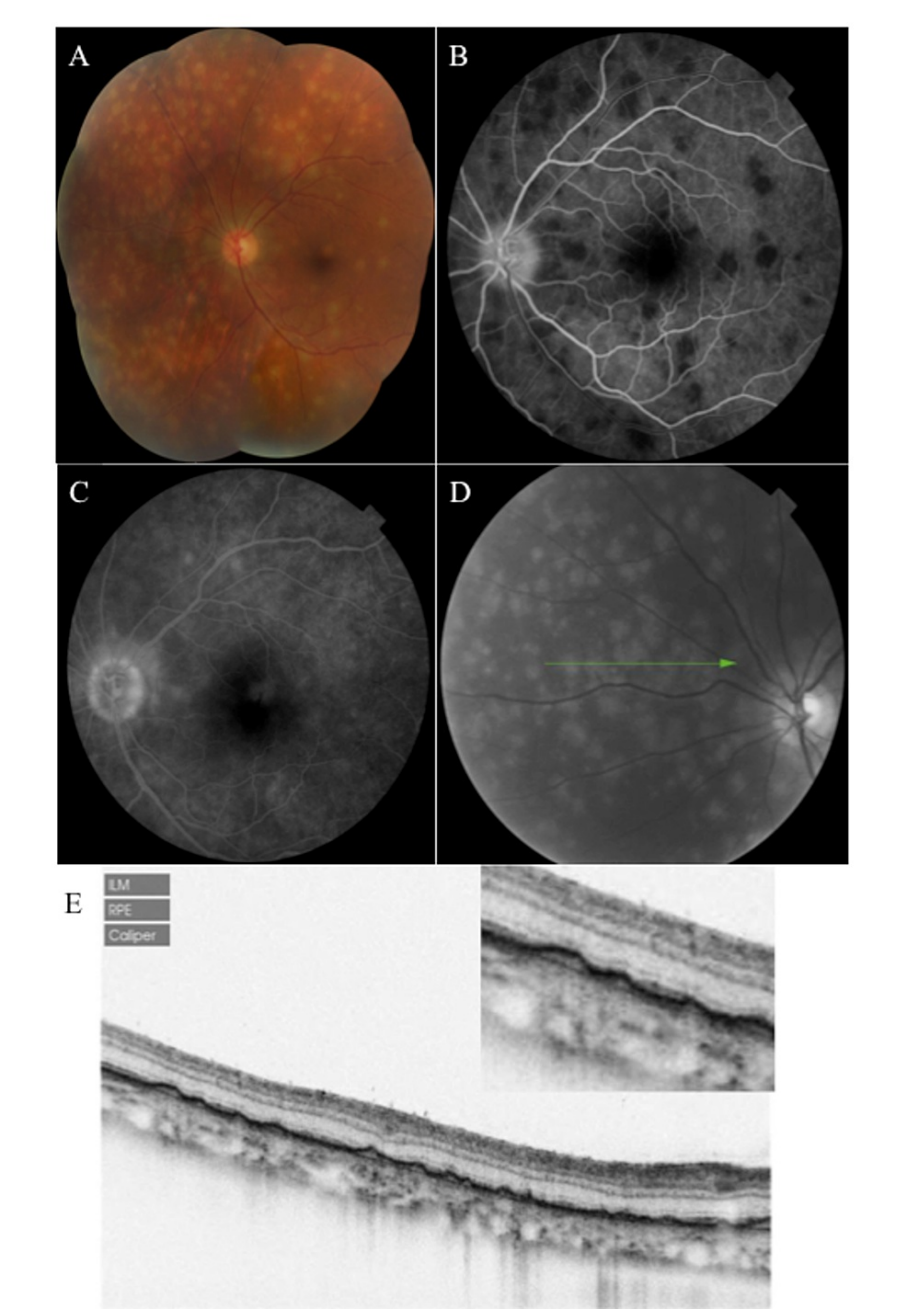
Visualisation of Dalén Fuchs nodules using various imaging modalities A: Multiple small yellow-white Dalén Fuchs nodules at the level of the retinal pigment epithelium. B: Dalén Fuchs nodules, hypofluorescent in *early* frames of fundus fluorescein angiography. C: Dalén Fuchs nodules, hyperfluorescent in *latter* frames of fundus fluorescein angiography. D: Spectral domain OCT through Dalén Fuchs nodules. E: Dome-shaped elevations of the retinal pigment epithelium, with Bruch’s membrane clearly seen as a separate hyperreflective layer underneath (blue arrows). OCT: Optical coherence tomography

Examination showed multiple small yellow-white Dalén Fuchs nodules at the level of the retinal pigment epithelium in the left eye as shown in Figure 1 (A). Figure 1 (B) reveals that the lesions were hypo fluorescent in early frames of fundus fluorescein angiography, but hyperfluorescent in the latter frames as seen in Figure 1 (C). Spectral domain optical coherence tomography (OCT) through Dalén Fuchs nodules, shown in Figure 1 (D), revealed dome-shaped elevations of the retinal pigment epithelium by underlying hyporeflective material as seen in Figure 1 (E). Bruch’s membrane was clearly seen as a separate hyperreflective layer underneath (blue arrows). Additionally, the choroid is seen infiltrated by hyperreflective cells mostly concentrated around the choroidal vessels.

A diagnosis of SO was made in light of clinical findings and imaging, with the OCT of the Dalén Fuchs nodule clinching the diagnosis. Since the focus of our report is on the diagnostic imaging findings, subsequent clinical management of the patient post-diagnosis has not been covered. 

## Discussion

Sympathetic ophthalmia is a rare condition and to date, there is limited imaging of the Dalén Fuchs nodules on OCT. While Correnti et al. [4] have produced OCT images of Dalén Fuchs nodules, their paper focused on a ‘likely’ case of SO. As such, these nodules have previously not been identified by OCT on a known case of SO. Additionally, there is no correspondence between the fundus photograph and the OCT.

It is also important to distinguish between SO and VKH, as both have similar case presentations including the development of Dalén Fuchs nodules [4]. VKH varies from SO based on patient history as the former does not present secondary to penetrating eye trauma or surgery, as was seen in this patient. Additionally, patients commonly present with optic nerve and choroidal involvement, as well as dermatological manifestations, none of which were present in the case featured.

In this case, OCT imaging has been key to identifying Dalén Fuchs nodules in a known case of SO. The imaging juxtaposed with clinical history and progression rules out other differential diagnoses such as sarcoidosis and tuberculosis and allows for a conclusive diagnosis of SO to be made.

## Conclusions

In our case, OCT imaging has been key to identifying Dalén Fuchs nodules in a known case of SO. We recommend that future research explore the possibility of the role of this diagnostic technique in the early detection of SO. 
